# With a little help from a friend – a new partner for acid-sensitive channels

**DOI:** 10.1007/s00424-026-03154-3

**Published:** 2026-02-13

**Authors:** Jochen Roeper

**Affiliations:** Institute for Neurophysiology, Neuroscience Center, Goethe University Frankfurt, Frankfurt am Main, Germany

The recent DPG paper of the month by Sven Kuspiel and colleagues from the Institute of Physiology at RWTH Aachen University features the discovery and characterisation of a novel interacting protein - *protein prenylcysteine oxidase 1 like* (Pcyox1l) with acid-sensitive ion channels (ASIC) [1].The team was headed by Stefan Gründer, who is a leading ASIC expert with an already extensive research tract on the molecular biophysics, pharmacology and function of this channel family (for review: [2]). In essence, they elegantly demonstrated that Pcyox11is a secreted protein that upon uptake into neurons acts as a binding partner to ASIC channels and promotes their surface expression and function.

## ASICs – before meeting PcyOX1L

ASICs (ASIC1-4 & splice variants) were initially homology cloned by the lab of Michel Lazdunski [3] based on their membership of the ENac/degenerin channel superfamily. The X-ray structure of ASIC channels consist of three subunits with a sodium preferring pore (Na/K permeability ratio: 3–12; [4]). Their defining functional feature is their fast activation by protons thus generating slowly desensitising (time constant 0.5–5 s) cationic currents in acidosis (pH < 7). In the nervous system, ASIC are thought to be mainly involved in acid-dependent pain processing by sensory neurons or hypercapnia-induced fear via neurons in the amygdala (for recent review: [5]). Given the exocytosis of synaptic vesicles also releases protons, ASIC also contribute – to varying degrees to excitatory postsynaptic currents (1–10% in CNS Synapses; >90% in the Merkel cell-afferent fibre synapse (see Fig. 13 in [5]). Given this contribution to postsynaptic currents, ASIC might also be involved in the control of synaptic plasticity in amygdala and hippocampus. Also, several pathophysiological roles in e.g. ischemia stroke and neuropathic pain have been proposed for ASIC (see Fig. 14 [5]).

## Pcyox1l – before meeting ASIC

The *protein prenylcysteine oxidase 1 like* (Pcyox1l) was largely uncharacterized until it was identified in a differential proteome screen comparing neutrophils from mice with Pseudomonas aeroginosa infection in the presence or absence of commensal colonization [6]. Crispr-mediated knockdown of Pcyox1l in granulocytes reduced their efficacy of killing Pseudomonas by more than 50%. Also, the pattern of protein prenylation (i.e. the transfer of farnesyl or geranylgeranyl moieties to cysteine residues on target proteins e.g. small GTPases) and the free farnesylcysteine and geranylgeranylcysteine metabolites during immune maturation of neutrophils were altered by the Pcyox1l knockout. In essence, Pcyox1l was identified as global modulator of the prenylation pathway in white blood cells in the context of trained immunity. Illustrating its fundamental cell biological roles [7] certain prenylation patterns are also required for hippocampal long-term plasticity [8] and ion channel trafficking [9].

## A new ASIC1- Pcyox1l partnership

The new study started with an in-depth proteomic analysis of ASIC1 interactors carried out by the company Logopharm in Freiburg (https://www.logopharm.com). They work in close collaboration with the lab of Bernd Fakler, who has specialized on identification, functional and structural characterisations of novel protein interactor of membrane proteins. Especially for AMPA-type glutamate receptors, this approach has resulted in a rich harvest of novel interactors that alter gating, biogenesis, trafficking and anchoring of this class of glutamate receptors (e.g [10, 11]). Solubilized native ASIC1 protein complexes from mouse brain were pulled down with two distinct ASIC1 antibodies (using brains from ASIC1knockout mice as controls) and subsequently analysed by mass spectrometry. As expected, this unbiased approach identified other know constituents of a heteromeric ASIC channel core complex –ASIC2 and ASIC4 subunits – with an about 1000-fold enrichment when purified from WT brains compared to using ASIC1 KO brains as source material. In the same category, only one other protein was discovered, which had no previously known relation to ion channels: the *protein prenylcysteine oxidase 1 like* (Pcyox1l) introduced above. Although Pcyox1l is robustly expressed in the brain (see https://www.proteinatlas.org/ENSG00000145882-PCYOX1L/tissue) and belongs to the same expression cluster as ASIC1 (brain cluster 22 – synaptic signal transduction (https://www.proteinatlas.org/humanproteome/tissue/expression+cluster#cluster22) its function for this organ has been unknown. The human protein database lists only two known protein-protein interactors with Pcyox1l including calnexin, a molecular chaperone in the ER that controls biosynthesis and trafficking of several ion channel complexes including voltage-gated potassium channels. Here, transient interaction with calnexin provides long-term protection of ion channel subunits from ER-associated degradation, facilitates maturation and membrane delivery [12].

Consistent with this database-inspired speculation (easy in hindsight) on the functional role of a Pcyox1l-ASIC1 interaction, the authors demonstrated a massive 5-fold increase of ASIC1-currents upon heterologous co-expression with Pcyox1l, independent of its potential enzymatic function. While all biophysical properties of ASIC1 where unaffected, membrane delivery of ASIC1 protein was clearly enhanced. Further studies revealed that Pcyox1l indeed facilitated the biogenesis of the trimeric ASIC complex, Next, the authors showed that shRNA-mediated knockdown as well as Pcyox1l overexpression altered native ASIC currents accordingly. Also, ASIC1 currents were significantly reduced in central neurons from heterozygous Pcyox1l KO mice. Together, these careful experiments clearly established a critical role of Pcyox1l for functional expression of native ASIC1. This finding alone, is a very important step towards a better understanding of the cell biology of ASIC.

Given that ASIC1 has been implicated in synaptic long-term potentiation (LTP; see above) the authors predicted that genetic inactivation of Pcyox1l would also reduce LTP, which indeed was the case. It remains to be shown that Pcyox1l does this solely via its interaction with ASIC1. It is unclear whether acute pharmacological inhibition of ASIC1 would occlude the effect of Pcyox1l inactivation on LTP induction and stability. Does overexpression of Pcyox1l boost LTP in a ASIC1-dependent way? These are potential future experiments to further define the exciting role of Pcyox1l in synaptic plasticity. Finally, the authors demonstrate the surprising fact that Pcyox1l does not need to be expressed in the same cell as ASIC to boost its function. They use very convincing co-culture experiments of Pcyox1l KO neurons with Pcyox1l WT glia to show that Pcyox1l is secreted by glia cells and taken up by neurons, where it is sufficient to boost ASIC surface expression and function. This novel glia-neuron collaboration in the context of synaptic signalling is reminiscent of other interactions regarding the activity-dependent glia release of D-Serine, a co-ligand of neuronal NMDAR essential for LTP [13]. Future studies might address whether glial Pcyox1l expression and secretion shares similar dynamics and activity-dependency.

In summary, this study demonstrates beautifully the power of an unbiased interactome analysis to expand our view on functional protein complexes, when it is combined with a wide-ranging and careful experimental validation strategy of a new and important protein-partnership. En route, the authors get slightly carried away by promoting Pcyox1l from crucial interacting protein to an indispensable subunit of ASIC1. Based on the given evidence, this might be an overinterpretation. Auxiliary channel subunits might be perceived as mostly monogamous in being exclusively dedicated to participating in a particular channel complex and co-regulating its core function of gating and ion flow. We must await the reverse purification of Pcyox1l to truly see how many protein interaction partners it does indeed possess in the brain.

## Data Availability

No datasets were generated or analysed during the current study.
